# Major Bleeding in the Emergency Department: A Practical Guide for Optimal Management

**DOI:** 10.3390/jcm14030784

**Published:** 2025-01-25

**Authors:** Sofia Bezati, Ioannis Ventoulis, Christos Verras, Antonios Boultadakis, Vasiliki Bistola, Nikolaos Sbyrakis, Othon Fraidakis, Georgia Papadamou, Barbara Fyntanidou, John Parissis, Effie Polyzogopoulou

**Affiliations:** 1 Department of Emergency Medicine, Attikon University Hospital, National and Kapodistrian University of Athens, 12462 Athens, Greece; christos.verras@gmail.com (C.V.); boult_doc@yahoo.gr (A.B.); jparissis@yahoo.com (J.P.); effiepol@med.uoa.gr (E.P.); 2 Department of Occupational Therapy, University of Western Macedonia, 50200 Ptolemaida, Greece; iventoulis@uowm.gr; 3 Second Department of Cardiology, Attikon University Hospital, National and Kapodistrian University of Athens, 12462 Athens, Greece; vasobistola@yahoo.com; 4 Department of Emergency Medicine, University Hospital of Heraklion, 71500 Crete, Greece; nikolasb@hotmail.com; 5 Department of Emergency Medicine, Venizelion Hospital of Heraklion, 71409 Crete, Greece; ofraidakis@gmail.com; 6 Department of Emergency Medicine, University Hospital of Larissa, 41334 Larissa, Greece; georgia.papadamou@yahoo.com; 7 Department of Emergency Medicine, AHEPA University Hospital, Aristotle University of Thessaloniki, 54636 Thessaloniki, Greece; bfyntan@yahoo.com

**Keywords:** major bleeding, haemorrhage, emergency department, practical guide, bundles of care, resuscitation

## Abstract

Major bleeding is a life-threatening condition with high morbidity and mortality. Trauma, gastrointestinal bleeding, haemoptysis, intracranial haemorrhage or other causes of bleeding represent major concerns in the Emergency Department (ED), especially when complicated by haemodynamic instability. Severity and source of bleeding, comorbidities, and prior use of anticoagulants are pivotal factors affecting both the clinical status and the patients’ differential response to haemorrhage. Thus, risk stratification is fundamental in the initial assessment of patients with bleeding. Aggressive resuscitation is the principal step for achieving haemodynamic stabilization of the patient, which will further allow appropriate interventions to be made for the definite control of bleeding. Overall management of major bleeding in the ED should follow a holistic individualized approach which includes haemodynamic stabilization, repletion of volume and blood loss, and reversal of coagulopathy and identification of the source of bleeding. The aim of the present practical guide is to provide an update on recent epidemiological data about the most common etiologies of bleeding and summarize the latest evidence regarding the bundles of care for the management of patients with major bleeding of traumatic or non-traumatic etiology in the ED.

## 1. Introduction

Major bleeding constitutes a main cause of visits to the Emergency Department (ED). Massive haemorrhage and critical bleeding are two distinct entities, not uniformly defined, which both carry considerable morbidity and mortality and require high vigilance from the clinician’s standpoint to initiate the appropriate treatment strategy in a timely manner. While massive haemorrhage refers to life-threatening bleeding leading to exsanguination and is associated with increased short-term mortality, critical bleeding denotes bleeding at a critical site which is related to dismal prognosis and long-term mortality. Early identification and control of the source of bleeding, coupled with assessment of the bleeding severity and the patient’s haemodynamic status, are the cornerstones in patient management that should guarantee optimal resuscitation.

This narrative review focuses on the holistic approach of the patient with major bleeding upon ED presentation in various clinical scenarios. The aim is to provide practical recommendations to physicians practicing in the ED setting, regarding identification, risk stratification, initial resuscitation and disposition of patients with major bleeding of various etiologies. Based on most recent guidelines published by distinct scientific groups and on latest evidence regarding resuscitation of patients with bleeding, we propose a roadmap for the management of patients with major bleeding from the ED perspective.

## 2. Definition

Currently, a universal definition of major bleeding is lacking [1]. Instead, diverse definitions can be found in contemporary literature, which stem from studies on patients with major trauma, massive gastrointestinal (GI) bleeding or bleeding associated with anticoagulant therapy, as well as from reports on massive transfusion protocols. Various terms have been used, such as “major”, “severe”, “critical”, “massive” or “life-threatening” bleeding/haemorrhage.

For instance, the International Society on Thrombosis and Haemostasis defines major bleeding as “fatal bleeding, and/or symptomatic bleeding in a critical area or organ, such as intracranial, intraspinal, intraocular, retroperitoneal, intra-articular or pericardial, or intramuscular with compartment syndrome, and/or bleeding causing a fall in haemoglobin levels of 2 g/dL or more, or leading to a transfusion of two units or more of whole blood or red blood cells (RBCs)” [2]. Patients with major trauma (defined as “significant injury or injuries that have potential to be life-threatening or life-changing sustained from either high energy mechanisms or low energy mechanisms in those rendered vulnerable by extremes of age” [3]) may also present with major bleeding warranting immediate resuscitation [4]. The latest European guidelines for the management of trauma patients [4] endorse the ATLS classification of blood loss [5] for the assessment of bleeding severity, even though recent evidence questions its clinical validity [6]. British guidelines for patients with upper [7] or lower [8] gastrointestinal bleeding give emphasis to the presence of haemodynamic instability, regarding it as the key characteristic for differentiating major haemorrhage. Although guidelines of the European Society of Gastroenterology do not include a definition for major haemorrhage, they recommend the application of risk stratification scores through the assessment of the haemodynamic status, which should be a basic feature in the primary investigation of patients with GI bleeding [9,10]. In studies on transfusion protocols, the most widely accepted definition of massive haemorrhage requiring massive transfusion (MT) is the administration of 10 units or more of whole blood or packed RBCs (pRBCs) within 24 h [11] or 5 units or more of RBCs within 4 h [12]; yet, the application of such a definition based solely on transfusion parameters is rather impractical in the ED setting, since it does not take into account the clinical status of the patient, which constitutes the core of medical practice in the ED.

Table 1 summarizes the terminology and definitions of critical/major bleeding, major/massive haemorrhage and massive transfusion, as proposed by several scientific societies, whereas Table 2 presents two novel metrics which have been developed in order to predict the need for massive transfusion and early mortality during resuscitation of patients with haemorrhage.

## 3. Epidemiology

Despite the fact that the need for urgent management of bleeding in trauma has been well documented and highlighted by the Golden Hour concept [19], epidemiological data on the ED management of non-traumatic bleeding remains scarce. In a small observational study, the main causes of major bleeding encountered in the ED were the following (in descending order of frequency): GI bleeding in 22%, intracranial haemorrhage (ICH) in 21%, haematuria in 19%, epistaxis in 18%, gynecological bleeding in 10%, haemoptysis in 7% and ocular bleeding in 4% of patients with major bleeding [20]. In another epidemiological study in the British population, the main causes of major bleeding were surgery (28.8%), obstetric bleeding (21.5%), gastrointestinal haemorrhage (20.2%) and traumatic bleeding (16.5%), while the overall 48-h mortality was 12% [21].

Trauma constitutes one of the leading causes of major bleeding, accounting for a significant proportion of ED visits and hospital admissions, and is responsible for 40% of preventable deaths worldwide [22]. When complicated by haemorrhagic shock, coagulopathy and multiple organ dysfunction syndrome, traumatic bleeding is associated with a mortality rate of approximately 12% [4] and necessitates prompt resuscitation through the implementation of a massive transfusion (MT) strategy [23]. Other causes of major bleeding that require the activation of an MT protocol include surgery [24,25], obstetric haemorrhage [26,27,28], GI bleeding [24,28,29], spontaneous bleeding in patients on anticoagulant therapy [30,31], and ruptured abdominal aortic aneurysm [28].

Obstetric haemorrhage remains a significant cause of morbidity and mortality worldwide, accounting for approximately 27% of maternal deaths [32]. Even in developed countries, mortality due to obstetric bleeding remains as high as 10% [33].

Mortality of upper GI bleeding is higher in patients with comorbidities, such as chronic kidney disease, malignancy, and alcohol abuse [34], ranging from 8 to 10% [35]. However, a recent systematic review reported a lower percentage of global case-fatality rates of upper GI bleeding (0.7–4.7%) [36]. In a UK audit, variceal upper GI bleeding accounted for 11% of hospitalizations and exhibited higher mortality than non-variceal upper GI bleeding. The proportion of patients presenting with some degree of haemodynamic instability [heart rate (HR) > 100 beats per minute (bpm)] was 22%, while approximately 14% of patients presented with hypotension and tachycardia [37]. In another clinical report for lower GI bleeding, mortality was lower (3.4%) and haemorrhagic shock had an incidence of 2.3% [38].

Regarding haemoptysis, the proportion of patients who develop massive bleeding is estimated to be in the range of 4.8–14% [39].

In patients on oral anticoagulant therapy, a large observational study reported that GI bleeding was the most common cause of ED presentation (34.5%), followed by minor trauma and epistaxis (20%), genitourinary bleeding (11%) and ICH (4%). Overall, nearly half of the patient population with anticoagulant-related bleeding required hospitalization [40]. Several studies have demonstrated the superiority of direct oral anticoagulants (DOACs) compared to vitamin K antagonists (VKA), with regard to their safety profile [41]. The incidence of major bleeding has been estimated to be between 4.6 and 6.7% in patients on DOAC therapy [42]. A systematic review and meta-analysis compared bleeding complications due to VKA and DOACs, and found that the use of VKA was associated with higher rates of major and fatal bleeding than the use of DOACs (4.64% versus 4% for major bleeding and 0.52% versus 0.30% for fatal bleeding, respectively [43]).

## 4. General Pathophysiological Concepts

Haemorrhagic shock is a type of hypovolemic shock due to blood loss, which leads to intravascular volume depletion, inadequate oxygen delivery and tissue hypoxia. According to the American College of Surgeons Committee on Trauma, haemorrhage can be classified into four stages with respect to the amount of blood loss [5]. In the initial stages I and II, homeostatic adaptive mechanisms are activated as a response to blood deficit, resulting in redistribution of blood flow and recruitment of capillary reserve, in an attempt to maintain tissue perfusion and aerobic metabolism. Should bleeding continue and exceed 30% of the estimated blood volume (stages III and IV), then changes occur at the cellular level, which include transition from aerobic to anaerobic metabolism with eventual derangements in electrolyte homeostasis and membrane integrity. Simultaneously, at the tissue level, hypovolemia and vasoconstriction result in diminished tissue perfusion and multi-organ damage, which in turn give rise to lactic acidosis, cellular necrosis, multi-organ failure, cerebral hypoxia, life-threatening arrhythmias and eventually death [28]. In parallel, systemic inflammatory response syndrome (SIRS) emerges as a consequence of tissue damage, while diffuse coagulopathy develops due to an imbalance between haemostatic and fibrinolytic mechanisms, which is characterized by a local hypercoagulant milieu at the site of bleeding on the one hand, and by inappropriate hyperfibrinolysis and endotheliopathy at the systemic level on the other [44]. All these derangements form a vicious cycle, which has been termed “lethal triad” and has been recently updated to the term “lethal diamond” with the addition of hypocalcemia to the existing triad of coagulopathy, acidosis and hypothermia. Although these pathophysiological derangements have been classically described in trauma patients [45,46], it should be pointed out that any form of bleeding resulting in haemorrhagic shock may exhibit similar abnormalities [44,47].

The main priority for the emergency physician managing patients with haemorrhage is to address specific questions in order to tailor initial resuscitation and timing of the appropriate intervention according to each clinical scenario. Thus, it is fundamental to follow a holistic approach, which should be based on the source, acuity and severity of bleeding, the haemodynamic status, the presence of coagulopathy, and the prior use of anticoagulant therapy. Throughout this holistic and systematic approach, control of bleeding, restoration of volume and blood loss, and reversal of coagulopathy remain the mainstays in the management of patients with major bleeding. Owing to its deleterious effects, diagnosis of haemorrhagic shock requires urgent management with rapid stabilization and source control. It should be noted that critical, non-massive bleeding, like intracranial haemorrhage, also requires immediate and focused resuscitation to prevent its detrimental clinical consequences [48].

## 5. Management

### 5.1. Initial Assessment

**Figure 1:**
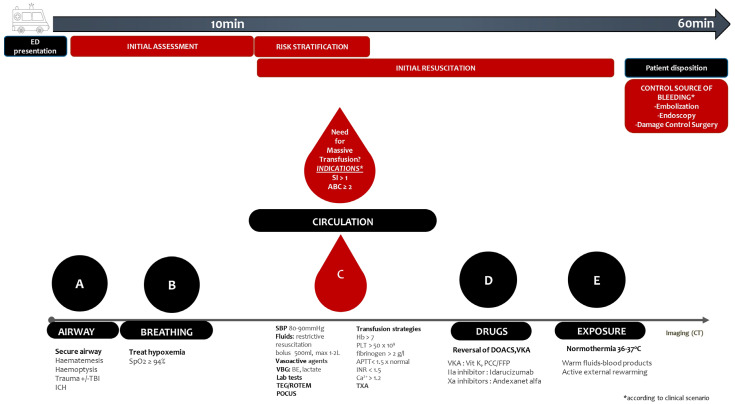
Roadmap for the management of patients with major bleeding in the ED. APTT = Activated Partial Thromboplastin Time; Ca^2+^ = Ionized Calcium; CT = Computed Tomography; DOACs = Direct Oral Anticoagulants; ED = Emergency Department; FFP = Fresh Frozen Plasma; Hb = Haemoglobin; IIa = Activated Factor II; ICH = Intracranial Haemorrhage; INR = International Normalized Ratio; PCC = Prothrombin Complex Concentrate; PLT = Platelets; POCUS = Point of Care Ultrasonography; SBP = Systolic Blood Pressure; SI = Shock Index; ROTEM = Thromboelastometry; TBI = Traumatic Brain Injury; TEG = Thromboelastography; TXA = Tranexamic Acid; VBG = Venous Blood Gas; Vit = Vitamin; VKA = Vitamin K Antagonists; Xa = Activated Factor X.

In the ED setting, initial assessment of patients with suspected or obvious bleeding should follow a systematic approach as part of a strategic therapeutic protocol. The primary goal of the initial assessment of the patient presenting with bleeding is to identify signs of haemorrhagic shock. Figure 1 depicts the roadmap for the management of patients with major bleeding in the ED.

#### 5.1.1. ABCDE Approach

Patients with non-traumatic haemorrhage should be evaluated through the ABCDE algorithm [49] in order to determine the haemodynamic status and the severity of haemorrhage. Initial evaluation should involve measurement of vital signs, including respiratory rate (RR), blood oxygen saturation (SpO_2_), systolic and diastolic blood pressure (SBP and DBP), heart rate (HR), capillary refill time (CRT), assessment of mental status and temperature. Important information, such as source of bleeding, pallor, mottled skin and clammy extremities, will also be retrieved during the stage of patient exposure [47]. Patients with upper GI bleeding may present with haematemesis, melena, or even haematochezia in cases where the haemorrhage is brisk and follows a rapid course through the GI tract. The most common presentation of lower GI bleeding is haematochezia [50]. Other causes of bleeding identified upon presentation are haemoptysis and vaginal bleeding.

For trauma patients, the ABCDE algorithm is modified into cABCDΕ, whereby “c” stands for catastrophic bleeding which needs to be immediately stopped either by local compression of open wounds or by applying a tourniquet for open extremity injuries. Foley catheter balloon tamponade has also been suggested as a measure to achieve prompt bleeding control [5].

Apart from physical examination, details regarding symptom onset, duration, frequency and volume of bleeding events, and other associated symptoms should be meticulously sought and documented. Patient history should also include information about comorbidities, such as cardiovascular, renal and liver disease, cancer and medication history. Special attention should be given to any prior use of non-steroidal anti-inflammatory drugs, corticosteroids, radiotherapy and drugs predisposing to bleeding, such as anticoagulants and antiplatelets, while the timing of the last dose administered should also be recorded.

#### 5.1.2. Blood Tests

Lactate and base deficit are useful biomarkers reflecting the degree of tissue hypoperfusion. Their measurement has been incorporated in the bundles of care for patients with trauma as a means of initially assessing blood loss severity and monitoring response to resuscitative interventions [4], while their assessment is also reasonable for patients with haemorrhagic shock of other etiologies [51]. In patients with GI haemorrhage and trauma, increased lactate levels upon ED admission have been associated with increased mortality [52,53,54,55] and the need for transfusion [56,57].

Essential laboratory tests include total blood count for measurement of haemoglobin (Hb), haematocrit (Hct) and platelets (PLT). Of note, an initial haemoglobin value within normal limits does not exclude severe bleeding, as in the initial stages of haemorrhage, when the patient has not yet received enough intravenous fluids, there is predominantly plasma volume loss, thus resulting in minimal changes in haemoglobin concentration [28]. A single measurement of haemoglobin is not accurate and may also be misleading; therefore, serial measurements are advocated as a more sensitive marker of bleeding severity [4]. Other valuable tests are coagulation parameters, including prothrombin time (PT), activated partial thromboplastin time (aPTT), international normalized ratio (INR) and fibrinogen, as well as renal function tests (urea and creatinine) and liver function enzymes. Blood typing and cross-matching should be performed in all instances, while prompt communication with the transfusion laboratory is mandatory to avoid turnaround time delays.

An alternative to traditional laboratory coagulation tests are viscoelastic tests (VET), namely thromboelastometry (ROTEM) and thromboelastography (TEG), which provide a real-time estimation of clot properties, speed of clot formation, clot stability and growth, and velocity of fibrinolysis. These tests have the advantage of depicting the patient’s coagulation status in a timely manner. Since severe bleeding may be rapidly complicated by coagulopathy, their application is of paramount importance in guiding transfusion of blood products [58]. They have been effectively used for correcting coagulopathy mainly in surgical [59] and trauma [60,61] patients, as evidenced by the fact their use has been associated with a decrease in both patient mortality and the need for excessive transfusion of clotting factors. As a result, their use has been embedded in the latest European guidelines for the management of trauma-induced coagulopathy [4]. Extending their application in other causes of major bleeding, like GI bleeding, seems appealing as an alternative to traditional laboratory testing [50,62], although more evidence is needed.

#### 5.1.3. Risk Stratification Scores

The application of risk stratification scores has been proposed as a means to estimate haemodynamic compromise, bleeding severity and/or the need for activation of an MT protocol. The most widely accepted score for bleeding severity is the classification of blood loss described by the American College of Surgeons, which incorporates vital signs (SBP, HR, and RR), mental status and biochemical variables (base deficit) in order to classify the amount of blood loss and determine the appropriate strategy of blood product transfusion. In other words, the degree of haemodynamic compromise dictates the need for transfusion, while severe haemorrhagic shock mandates the activation of an MT protocol, as part of the initial resuscitation [5]. Emphasis should be placed on the fact that hypotension is not a sensitive marker of haemodynamic instability per se, given that the activation of compensatory neurohormonal and cardiovascular mechanisms may counterbalance blood loss and mask the presence of haemorrhagic shock, in such a way that hypotension may become evident only when blood loss has exceeded 30% of the estimated blood volume [44].

Unfortunately, there are no universal criteria for the activation of an MT protocol in patients with severe haemorrhage [11]. Clinical gestalt or various proposed risk stratification scores exhibit moderate sensitivity and specificity; thus, more research is needed to cover this gap regarding the emergency treatment of every single patient with major bleeding [63].

In trauma patients, Shock Index (SI = HR/SBP, normal range 0.5–0.7) is a simple and practical metric that can be easily applied at bedside, upon ABCDE assessment to determine shock severity. Its use has been recommended by recent European guidelines on management of major bleeding in trauma patients [4]. An SI > 0.91 is predictive of both the need for massive transfusion [64] and increased mortality [65]. The Assessment of Blood Consumption (ABC) score, which includes history of penetrating trauma, SBP < 90 mmHg, HR > 120 bpm, and positive Focused Assessment with Sonography in Trauma (FAST), has also been shown to predict massive transfusion [66,67], although recent data question its reliability [68].

In non-trauma patients, data regarding the initiation of an MT protocol are scarce and widely heterogeneous [1]. Nevertheless, patients with severe haemorrhage and haemodynamic instability may benefit from the activation of an MT protocol, since it may prevent inappropriate transfusion delays [69]. Likewise, the application of SI upon presentation is recommended by the British Society of Gastroenterology for the risk stratification of patients with lower GI bleeding to identify those patients who will benefit from an urgent computed tomography angiography (CTA) and interventional radiology management [8]. SI may also be a useful tool in patients with upper GI bleeding, as it has been found to be associated with increased mortality [70], while it also seems to predict the need for prompt endoscopic therapy, hospitalization in the intensive care unit, and RBC and blood component transfusion [71]. In patients with non-variceal upper GI bleeding, the Glasgow-Blatchford Bleeding Score (GBS) [72] is recommended to identify low-risk patients who could be possibly treated as outpatients. In women with postpartum haemorrhage, SI is endorsed by the International Federation of Gynecology and Obstetrics for the assessment of bleeding severity [73].

### 5.2. Resuscitation

**A.**  **Airway**

Special consideration for airway protection with endotracheal intubation is warranted in unstable patients with GI bleeding in case of protracted haematemesis or altered mental status [9,74], in patients with massive life-threatening haemoptysis [39,75], and in patients with suspected intracerebral haemorrhage (ICH) and altered level of consciousness [48]. In trauma patients, criteria for intubation are airway obstruction, decreased level of consciousness or compromised ventilation and oxygenation [4]. A clinical decision for intubation should be preceded by adequate volume resuscitation in order to avoid cardiovascular collapse due to low cardiac output. Therefore, a delayed intubation strategy with haemodynamically safe induction agents is preferred so as to allow for adequate haemodynamic support of the patient before intubation, with administration of fluids, blood products and vasopressors [76,77,78].

**B.**  **Breathing**

Oxygen supplementation should be provided as part of the initial resuscitation in case of hypoxemia [4,74]. Normocapnia is the desired target when adjusting ventilation parameters in patients with intracranial traumatic or non-traumatic haemorrhage [4,48].

**C.**  **Circulation**

Patients with signs of haemodynamic compromise should be immediately stabilized. The first goal is the restoration of intravascular volume as a means to counteract tissue hypoxia induced by hypoperfusion. The concept of permissive hypotension [target SBP 80–90 mmHg] is widely endorsed in patients with traumatic and non-traumatic massive haemorrhage [4,5,39,73,74,79,80]. However, in patients with severe traumatic brain injury with Glasgow Coma Scale (GCS) < 8, it is recommended that mean arterial pressure (MAP) should be maintained above 80 mmHg [4].

Regarding patients with suspected ICH, the target SBP should be 130–140 mmHg, which needs to be achieved gradually within an hour and maintained thereafter in order to improve functional status and prevent haematoma expansion [48]. The recommended agents for SBP control are labetalol, nicardipine and hydralazine administered intravenously [81].

***C1.***  ***Fluids***

The initial step is administration of crystalloid fluids (0.9% sodium chloride or balanced crystalloid) as fluid boluses of 500 mL, while following a restrictive fluid resuscitation strategy (maximum volume of 1–2 L) [4,7,9,82]. Liberal fluid administration has been associated with volume overload, coagulopathy, multi-organ failure, acute respiratory distress syndrome, increased need for blood product transfusion and mortality in trauma and surgical patients [83,84,85,86]. The choice of the appropriate type of fluid is still a matter of debate; yet, evidence favors the use of balanced crystalloids, given the fact that saline infusions may lead to hyperchloremic acidosis, acute kidney injury and increased mortality [87,88]. In case of traumatic brain injury, administration of Lactated Ringer’s solution should be avoided [4].

***C2.***  ***Vasoactive agents***

If fluids are inadequate to stabilize the haemodynamic status of patients with traumatic bleeding, initiation of vasoactive agents is indicated to maintain blood pressure at target levels. Noradrenaline is the vasopressor of choice and dobutamine may be considered in case of myocardial dysfunction [4]. In patients with GI bleeding, there is a paucity of data regarding the administration of vasopressors in case of haemodynamic instability. Of note, only a small randomized controlled trial (RCT) demonstrated that haemodynamically unstable patients with non-variceal upper GI bleeding who received restrictive fluid resuscitation in combination with dopamine had better prognosis compared to patients receiving aggressive fluid resuscitation [89]. An Expert Consensus Statement about the management of patients with GI bleeding [74], embracing guidelines on perioperative bleeding [90], recommends early use of noradrenaline to maintain MAP above 65 mmHg, if fluids and pRBCs fail to restore haemodynamic instability. For patients with variceal bleeding, administration of terlipressine, somatostatin or octreotide is indicated, as they cause splanchnic vasoconstriction [7,90].

***C3.***  ***Transfusion strategies***

***(i)***  ***Massive transfusion protocols***

Patients with life-threatening bleeding are candidates for an MT protocol, which practically streamlines the disposition of blood components and products in a predefined fixed ratio. It is a multidisciplinary task designated to guarantee administration of transfusion products within the first 15 min upon recognition of massive haemorrhage [91]. Implementation of an MT strategy has successfully decreased mortality in patients with major trauma [92,93]. However, criteria for the activation of an MT protocol are neither well defined nor universal. Therefore, its activation is currently driven by clinical gestalt, risk stratification scores or regional guidelines in a variable pattern [94]. In order to set the alarm for an MT protocol, the Canadian Blood Services Society [95] and the Australian Patient Blood Management Guidelines [14] recommend the use of one or more of the following objective scores: CAT (Critical Administration Threshold, SI (Shock Index), ABC (Assessment of Blood Consumption) or RABT (Revised Assessment of Bleeding and Transfusion). Other than major trauma, patients with upper GI bleeding and postpartum haemorrhage complicated by haemodynamic instability also require prompt activation of an MT protocol [7,73].

The majority of evidence regarding MT derives from databases of trauma patients [94]. Based on the results of a rather recent RCT, administration of blood products in a 1:1:1 (plasma:platelets:pRBCs) ratio is advocated in order to minimize dilutional coagulopathy and achieve haemostasis [96]. However, a transfusion strategy of 1:1:2 ratio is also acceptable, since comparable outcomes have been reported with regard to mortality [97]. In non-trauma patients with massive haemorrhage, a 1:1:1 ratio has not demonstrated clear benefit [29,69,98,99], hence a lower ratio 1:1:2 may be more appropriate [100]. Patients with severe GI bleeding should be promptly resuscitated according to MT protocols with a 1:2 rather than 1:1 plasma:RBC ratio, although more research is needed to clarify which regimen yields better outcomes [1,101,102]. The European Society of Intensive Care Medicine makes no recommendation for or against empirical transfusion strategies at fixed ratios, due to paucity of evidence in non-trauma populations. Nevertheless, the scientific committee acknowledges the necessity for an urgent intervention in patients with massive haemorrhage and underscores the need for a coordinated and organized transfusion protocol in non-trauma populations as well [103]. Empirical initiation of an MT protocol should be considered as a means to replace blood loss in a manner that resembles whole blood composition, in terms of RBCs, clotting factors and platelets [58]. In addition, transfusion of blood products should be performed through a warming device to avoid exacerbation of hypothermia [13].

***(ii)***  ***Goal directed transfusion strategy***

1. Address anemia

**Packed Red Blood Cells.** Transfusion of pRBCs is mandatory to restore the oxygen debt and ameliorate oxygen carrying capacity. Transfusion of pRBCs is indicated in patients with trauma [4], GI bleeding [7,9,10,66] and postpartum haemorrhage [73], following a restrictive transfusion strategy at a haemoglobin threshold of 7 g/dL, except for patients with coronary artery disease who will benefit from a higher transfusion threshold of 8 g/dL to avoid myocardial ischemia. It needs to be noted that levels of haemoglobin should not be the only trigger for initiating transfusion in critically ill bleeding patients, if haemodynamic instability is present. Clinical haemodynamic parameters should be consistently assessed to guide decision-making [104,105]. The concept behind readily available uncrossmatched RBCs (URBCs) for transfusion, already from the ED [106], lies in the observation that for every 1 min of delay in blood delivery, mortality increases by 5% [91]. Therefore, transfusion of URBCs is encouraged in the haemodynamically unstable patient with active life-threatening bleeding [1,107,108].

2. Address coagulopathy

Trauma-induced coagulopathy has been well described in the literature [45] and early transfusion of clotting factors and platelets has been shown to reduce mortality in patients with trauma [96,109]. Patients with upper GI bleeding may also exhibit various degrees of coagulation abnormalities due to haemorrhage severity [110] and liver disease [111]. In a UK audit, a large proportion (16.4%) of patients with upper GI bleeding had an INR > 1.5 and nearly half of those (46%) were receiving anticoagulant therapy [37]. Moreover, a secondary analysis of the aforementioned UK audit, including only patients with nonvariceal upper GI bleeding, reported that patients with coagulopathy on presentation had increased risk of haemorrhagic shock and mortality [110]. Regarding patients with lower GI bleeding, 10.6% had an INR > 1.5 and the majority of those (82%) were under treatment with anticoagulants, while 1% had liver disease [38]. Patients with massive bleeding may experience coagulopathy due to large amounts of blood loss (consumptive coagulopathy) or after administration of large amounts of fluids and/or RBCs (dilutional coagulopathy). Moreover, metabolic acidosis due to hypoperfusion and hypothermia contributes to coagulation abnormalities [112]. Patients with haemorrhagic shock of various etiologies may suffer from systemic derangements in the coagulation status as a result of sympathoadrenal activation and endotheliopathy; a condition defined as shock-induced endotheliopathy (SHINE) [113]. Accordingly, haemostatic resuscitation with clotting factors and platelets is strongly encouraged.

**Fresh Frozen Plasma (FFP).** Fresh frozen plasma (FFP) has been traditionally used to reestablish balance in the consumption of clotting factors in patients with active bleeding, based on prolonged laboratory values of PT/INR/APTT. It contains all clotting, anticoagulant and antifibrinolytic factors at a concentration of 0.5–1.0 IU/mL [114], as well as albumin and immunoglobulins [115]. It is noteworthy that the concentration of fibrinogen and clotting factor VIII in a unit of FFP (250 mL) is variable, thus rendering its use as a replacement therapy of fibrinogen levels unreliable [104]. The indication to initiate FFP administration is a PT/INR and/or APTT value 1.5 times above the normal limits, with the usually recommended dose being 15–20 mL/kg (4–6 units) [13,116,117], although the essential FFP dose for achieving an increase in fibrinogen levels by 100 g/L is actually 30 mL/kg [118]. Holland et al. have proposed a practical equation to predict the increase in INR per unit of FFP administered, based on the value of pretranfusion INR. According to this equation, the INR change should be equal to *0.37 [pretransfusion INR] − 0.47*; r^2^ = 0.82 [119]. Nevertheless, administration of large volumes of FFP has been associated with various complications, such as transfusion-associated cardiac overload (TACO), transfusion-related lung injury (TRALI), and allergic reactions [104,120].

**Prothrombin Complex Concentrate (PCC), Cryoprecipitate and Fibrinogen Concentrate (FC).** The use of coagulation factor concentrates, such as prothrombin complex concentrate (PCC), cryoprecipitate, fibrinogen concentrate (FC) and factor XIII concentrate (FXIII), has been introduced as an alternative treatment to prevent complications from transfusion of large volumes of FFP [13]. Their administration should ideally be guided by the results of VET analysis [58]. Four vitamin K-dependent coagulation factors (activated factors II, VII, IX and X), protein C, and protein S [114] are contained in 4-factor PCC. In comparison to FFP, 4-factor PCC has a substantially higher concentration of clotting factors; as a matter of fact, it contains 25 times the amount of clotting factors found in FFP (25 IU/mL versus 0.5–1.0 IU/mL) [114]. Practically, in terms of volume administration, the amount of clotting factors found in a dose of PCC (20 mL) corresponds to 500–1000 mL of FFP (2–4 units). Another advantage of PCC is that it has a more immediate effect, as it leads to the desired INR reduction more rapidly [121]. Although there is an obvious need to investigate the exact clinical benefit of PCCs in other clinical scenarios beyond trauma, administration of PCCs under the guidance of laboratory testing/VET analysis is reasonable in all patients with coagulopathy.

Cryoprecipitate contains factor XIII, factor VIII:C, fibrinogen, von Willebrand factor (VWF), and fibronectin. The standard dose is 10 units (each unit = 15–20 mL) and is expected to raise fibrinogen levels by approximately 1 g/L [13]. Alternatively, fibrinogen concentrate (FC) at a dose of 25–50 mg/kg can be administered depending either on VET results or on low fibrinogen plasma levels [74]. The recommended dose by the producer is 70 mg/kg with the advice to monitor clotting times and re-administer as indicated [122]. FC has been approved for supplementation of fibrinogen levels solely in bleeding patients with congenital hypofibrinogenemia or afibrinogenemia. Recently, the encouraging results of the FIBRES study [123] led to the approval of FC administration in bleeding patients with acquired hypofibrinogenemia by the United States Food and Drug Administration (FDA) [124]. As in the case of PCCs, despite the fact that clinical evidence is lacking, there is a clinical rationale for the administration of FC in patients with massive haemorrhage and documented low levels of fibrinogen [125,126].

**Platelets.** Platelet transfusion is indicated in patients with active bleeding and thrombocytopenia (<50 × 10^9^/L) [13]. For patients with traumatic or spontaneous ICH, platelet count should be maintained above 100 × 10^9^/L [127].

**Strategies to reverse coagulopathy in different clinical scenarios**: Regarding trauma patients with massive haemorrhage, initial haemostatic resuscitation should include blind administration of either FC/cryoprecipitate or FFP in conjunction with pRBCs. Fibrinogen should be given at an initial empiric dose of 2 g in order to reverse hypofibrinogenemia.

Additional supplementation of clotting factors, through administration of FFP or PCC, should be guided by objective evaluation of coagulopathy, as evidenced by laboratory measurement of clotting times (PT and/or APTT 1.5 times above normal values) or VET results indicative of depletion of specific factors. Correction of hypofibrinogenemia should be based on abnormal VET results or on low fibrinogen plasma levels (below 1.5 g/L), and it should be performed either with a dosage of 3–4 g of FC or with 15–20 units of cryoprecipitate, and not with FFP [4,128].

Platelets should be administered as part of the initial blind resuscitation strategy in patients with active haemorrhage in combination with FC/cryoprecipitate or FFP and pRBCs. Ongoing supplementation should be based on platelet count with a threshold of <50 × 10^9^/L or <100 × 10^9^/L in patients with traumatic brain injury [4,13].

In contrast to the extensive recommendations on the management of trauma patients, data regarding appropriate haemostatic resuscitation are scarce in patients with GI bleeding. In cases of active bleeding, the evaluation of coagulation defects should be based on documented low fibrinogen levels (<1.5–2.0 g/L) and/or low platelet count (<50 × 10^9^/L) and/or VET results, while eventual correction of any coagulation abnormality should be performed by supplementing the corresponding deficient coagulation factor [101]. The United Kingdom’s National Health Service (NHS) in a recent guidance document suggests the adoption of more restrictive strategies regarding transfusion of plasma and platelets in patients with GI bleeding [102].

Increased vigilance is required in patients with liver failure, since its presence has been associated with increased incidence of massive GI bleeding. These patients comprise a distinct population presenting with a mixed phenotype of procoagulation and anticoagulation defects [101]. Conventional laboratory testing for the assessment of coagulation status (INR, PT, APTT, fibrinogen and platelet levels) is of low clinical utility, as patients typically exhibit abnormal values [101,111]. VET-guided transfusion of coagulation factors with PCC and FC is preferred over FFP so as to avoid elevations in portal pressure [74,129]. Special caution is warranted in patients with cirrhosis and active variceal bleeding, given the fact that FFP has been associated with deterioration of portal hypertension [130], increased mortality, inadequate bleeding control and prolonged hospitalization [90].

In patients with postpartum haemorrhage, attention should be paid to the early administration of fibrinogen, since its levels normally rise to 4–6 g/L in the third trimester of pregnancy [126]. Consequently, the threshold for administering fibrinogen is slightly higher, at 2 g/L [73].

While emerging evidence favors management of coagulation defects guided by VET studies which yield more rapid results, their availability is not widespread. Therefore, in healthcare settings with limited resources, clinicians should tailor their management protocols according to available local resources and develop a strategic plan that incorporates close communication with the laboratory, which will enable them to obtain early results of clotting times and thus guide appropriate treatment in a timely manner.

***C4.***  ***Supplemental therapies to blood products***

***(i)***  ***Calcium supplementation***

Attention should be paid to plasma ionized calcium concentration. Calcium is an essential factor participating in various stages of the coagulation cascade. Thus, hypocalcemia should be adequately managed to minimize coagulation defects and prevent cardiac dysrhythmias. Transfusion-induced hypocalcemia (<0.9 mmol/L) is the result of citrate-mediated chelation of serum ionized calcium. The trigger to administer calcium in the form of calcium chloride 10% (270 mg of elemental calcium/10 mL) is plasma ionized calcium below 1.2 mmol/L (0.5–1.0 g intravenously for every 500 mL of transfused blood) [74].

***(ii)***  ***Tranexamic acid***

Tranexamic acid is an antifibrinolytic agent which can be used as adjuvant treatment to counteract hyperfibrinolysis [131]. Two large randomized clinical trials have demonstrated its beneficial effect in trauma patients. The CRASH-2 study showed a 1.5% reduction in mortality in patients with extracranial traumatic bleeding [132], while the CRASH-3 study reported a mortality benefit only in patients with mild or moderate traumatic brain injury [133]. As a result, European guidelines on the management of major bleeding in trauma patients strongly recommend its early administration within the first 3 h of injury at a dose of 1 g over 10 min, followed by the infusion of 1 g for 8 h thereafter [4].

The role of tranexamic acid is more equivocal in patients with GI bleeding. Recently, the HALT-IT study reported no mortality benefit in patients with severe GI bleeding receiving a high dose of tranexamic acid (1 g as a loading dose and 3 g over a period of 24 h). Furthermore, the risk of venous thromboembolic events and seizures was increased, hence discouraging the administration of tranexamic acid in patients with GI bleeding [134]. However, in patients with liver failure, the use of tranexamic acid may be considered if hyperfibrinolysis has been documented by the results of VET analysis [111,129]. Administration of tranexamic acid is also recommended in patients with massive haemoptysis, although its use is supported mainly by RCTs on patients with non-massive haemoptysis [135]. It may be administered either through nebulization at a dose of 500 mg every 8 h or intravenously at an initial dose of 1 g and subsequently at an infusion rate of 1 g over a period of 8 h. The rationale behind its administration in this patient population is that its use may lessen both the duration and the severity of bleeding, and also reduce the frequency of transfusions and invasive interventions [39]. Additionally, due to its safety profile and favorable effects on maternal survival, the use of tranexamic acid is supported in patients with postpartum haemorrhage [73,136,137].

Table 3 summarizes the management of major bleeding in different clinical scenarios.

**D.**  **Drugs—Reversal of antithrombotic agents**

***D1.***  ***Anticoagulants***

Current practice on the use of anticoagulation therapy favors the administration of direct oral anticoagulants (DOACs) instead of vitamin K antagonists for the management or prevention of thrombosis. Despite the fact that DOACs have reduced the risk of haemorrhagic events, a substantial number of patients may still present to the ED with major bleeding [139]. Reversal of their anticoagulant effect is of paramount importance in case of bleeding at a critical site, or in the event of haemodynamic instability and/or excessive bleeding resulting in ≥2 g/dL drop in haemoglobin levels or requiring ≥ 2 units of pRBCs [140].

***(i)***  ***Direct oral anticoagulants (DOACs)***

DOACs exert their anticoagulant effects through direct binding and inhibition of factor IIa (dabigatran) or factor Xa (apixaban, rivaroxaban, endoxaban and betrixaban). Certain issues should be addressed before managing a patient on DOAC therapy, such as the type of agent, the time since last dose intake, the half-time and the mode of excretion of the drug, as well as the presence of specific comorbidities, like renal impairment.

Strategies to minimize the anticoagulant effect should be focused on rapid excretion of the drug and pharmacodynamic reversal. Thus, if the time since last dose intake is less than 2 h, administration of activated charcoal is recommended to prevent further absorption of the drug [140,141]. Regarding dabigatran, haemodialysis could be considered to remove the residual drug from the circulation [140,142]. However, in the cases of apixaban and rivaroxaban, haemodialysis is not an effective therapeutic option, as both agents are highly protein-bound [139].

Considering that DOAC-treated patients presenting with major bleeding require urgent and effective therapeutic actions, the mainstay of their management is the administration of specific antidotes to reverse the effect of DOACs [141,143]. Idarucizumab is a monoclonal antibody that binds dabigatran with high affinity and specificity and results in its rapid reversal within minutes, at a dose of 5 g intravenously. It should be noted that, due to its high affinity for dabigatran, it does not exert prothrombotic effects [141]. Since 2016, it has been approved for patients with life-threatening haemorrhage (ICH or other critical site bleeding and/or expanding or uncontrolled bleeding), as well as for patients with an urgent indication of surgery [144]. Andexanet alfa is a modified human Xa protein that binds Xa inhibitors, thereby neutralizing their effect. It has exhibited encouraging results on haemostatic efficacy, although a slight increase in thrombotic events has been reported [143]. Accordingly, its use has been approved since 2019 for patients with life-threatening or major bleeding (ICH or GI bleeding), provided that it is administered within 18 h from the anticoagulant’s last dose. Two dose regimens are available, depending on the specific Xa inhibitor used (apixaban, rivaroxaban or andoxaban), as well as on the dosage and the timing of the last dose [141].

If reversal agents for dabigatran or direct Xa inhibitors are not available, supportive treatment with 4-factor PCC is indicated at a dose of 25–50 IU/kg (max 4000 IU) or 2000 IU at a fixed dose, with the rationale of indirectly counteracting their anticoagulant effect [140]. This alternative could be mainly applied in low-income settings, especially when taking the substantial cost of the antidote into consideration.

***(ii)***  ***Vitamin K antagonists***

Warfarin acts by inhibiting synthesis of vitamin K-dependent clotting factors (II, VII, IX, X), and has a long half-life of approximately 35 h [145]. Therefore, in warfarin-treated patients with major bleeding, the administration of vitamin K (the specific reversal agent of warfarin) is required, combined with administration of an immediate source of clotting factors. It needs to be emphasized that vitamin K permits synthesis of new clotting factors, but has a late onset of action. The recommended regimen includes slow (10–30 min) intravenous administration of vitamin K, in conjunction with plasma or PCC, which should result in INR reduction after 4–6 h. The dose of both vitamin K and PCC should be based on the baseline INR value [140]. PCC is favored over FFP, since the use of the latter may be accompanied with adverse effects and delays related to plasma thawing and ABO cross-matching [146].

***(iii)***  ***Unfractionated heparin (UFH), low-molecular-weight heparin (LMWH) and fondaparinux***

Unfractionated heparin (UFH) inactivates thrombin and factor Xa through binding to antithrombin III, while low-molecular-weight heparin (LMWH) indirectly inhibits factor Xa. The reversal agent for UFH or LMWH is protamine and should be provided in a dose- and time-dependent manner. The anticoagulant effect of fondaparinux may be reversed by recombinant activated factor VII or activated PCC (aPCC), albeit with a low quality of evidence [139].

Table 4 summarizes the reversal of anticoagulant agents in patients with major bleeding.

***D2.***  ***Antiplatelet agents***

In cases of acetylsalicylic acid or adenosine diphosphate (ADP) inhibitors, potential risks from reversal of their antiplatelet activity should be weighed over the benefits of their continuation, especially when patients have ischemic heart disease. Thus, in patients with GI bleeding, aspirin should not be routinely withheld, especially when administered as monotherapy for secondary cardiovascular prevention. However, if bleeding is life-threatening, aspirin should be discontinued and resumed as early as possible, ideally 3–5 days after having achieved successful haemostasis. In cases of dual antiplatelet therapy, the second agent should be discontinued on an individualized basis. Routine platelet transfusion is not recommended [9,10,147]. Furthermore, two recent systematic reviews showed that platelet transfusion had a neutral survival effect in patients with traumatic or spontaneous ICH who were on antiplatelet therapy [148,149]. A potential exception could be for patients with ICH who are candidates for urgent neurosurgical treatment, in whom platelet transfusion may be considered if there is coexisting thrombocytopenia. The supportive administration of desmopressin has not demonstrated any clear benefit, hence its use is not encouraged [48,150].

**E.**  **Exposure**

Hypothermia should be invariably managed in all patients with life-threatening bleeding, regardless of the etiology. Given the fact that hypothermia exerts deleterious effects on coagulation and platelet function and results in the development of acidosis [151], it is recommended to consistently pursue normothermia at 36 °C–37 °C. Heat loss should be prevented by removal of any wet clothes and application of appropriate blankets. Further measures to prevent and treat hypothermia include transfusion of fluids and blood products through a warming device [13,152] and application of active warming through forced warm air [4,153].

### 5.3. Imaging

#### 5.3.1. Point of Care Ultrasonography (POCUS)

Bedside ultrasonography is a useful tool which not only provides important information about the haemodynamic status of patients with haemorrhage, but also aids in the detection of the source of bleeding. Due to its flexibility, it allows real-time assessment of the patient’s volume status and thereby should be performed not only upon patient presentation but also throughout the course of resuscitation [154].

In patients with undifferentiated shock, a Rapid Ultrasound in Shock (RUSH) protocol [155] may aid in the detection of hypovolemic shock [156,157]. For instance, a hypercontractile heart in conjunction with a collapsible inferior vena cava are signs of hyperdynamic circulation indicative of volume depletion, which, in the context of occult or obvious bleeding, strengthens the diagnosis of haemorrhagic shock.

Moreover, POCUS may reveal the cause of bleeding in trauma patients or guide further diagnostic work-up in case a ruptured abdominal aneurysm, ovarian cyst or ectopic pregnancy is suspected. In patients with thoracoabdominal injuries, a FAST protocol is strongly recommended for the identification of free fluid in the abdominal, pericardial and pleural space [4,5].

#### 5.3.2. Computed Tomography (CT)

Contrast-enhanced computed tomography (CT) or CT angiography (CTA) is the gold standard examination for the diagnosis of active bleeding in all clinical cases. Upon patient stabilization and according to the clinical scenario, a CT should be performed to resolve diagnostic uncertainties.

Many retrospective observational analyses on trauma patients report that contrast-enhanced whole body computed tomography (WBCT), when compared to selective CT, is associated with better survival in severely injured patients [158] with haemodynamic instability [159] or in patients with traumatic brain injury and altered level of consciousness (GCS < 12) [160]. Beyond the survival benefit, WBCT reduces the time to final diagnosis [161] and the length of stay in the ED [162], although it should be reserved for patients with clinically significant injury and/or altered haemodynamics and/or altered level of consciousness and/or severe mechanism of injury [4,163].

Patients with lower GI bleeding and haemodynamic instability should undergo CTA to identify the site of bleeding for diagnostic and therapeutic purposes [8,10]. Patients with massive haemoptysis who are haemodynamically compromised should undergo contrast-enhanced CT or CTA in order to diagnose the site of bleeding, if bronchial arteriography with embolization is not readily available [164].

For patients with suspected ICH, a CT scan should be performed to exclude ischemic stroke or other intracranial pathology. CTA is further recommended for selected patients based on site of ICH, age and comorbidities in order to determine underlying pathology and tailor appropriate therapy [48].

## 6. Future Perspectives

Novel emerging techniques could enhance the management of patients with major bleeding. Artificial intelligence could aid in the risk stratification of patients with bleeding through the application of machine learning algorithms. Compensatory reserve index (CRI) is a novel non-invasive tool based on a machine learning framework, which has been proposed as a potentially useful method of mirroring the extent of haemorrhagic shock and predicting the need for blood product transfusion in trauma patients [165]. Moreover, according to a systematic review regarding the use of various machine learning tools as predictors of outcomes in patients with GI bleeding, artificial neural networks (ANN) exhibited a good prognostic performance in predicting blood transfusion, need for intervention and mortality, compared to the Glasgow-Blatchford and the Rockall clinical scores with a median AUC of 0.93 (range 0.78–0.98) [166]. Although artificial intelligence tools seem to be promising, risk stratification tools require external validation in randomized clinical studies in order to be incorporated in the future management of patients with major bleeding.

Additionally, ongoing research focuses on both already existing and novel pharmacologic interventions regarding transfusion of blood components and supplemental therapies. To this end, administration of concentrated factors, like recombinant factor VIIa and factor XIII [167], as well as cold stored platelets [168], have shown encouraging results in certain populations, but their effects should be further evaluated. Given the well-recognized beneficial effect of tranexamic acid, other antifibrinolytic agents, like aprotinin, epsilon-aminocaproic acid [167], and derivatives of 1,2,3-triazole [169], are under investigation. Furthermore, large-scale randomized trials should delineate the role of desmopressin in patients with major bleeding [170].

## 7. Conclusions

Major bleeding is a life-threatening condition with inherently high mortality rates. Lessons from the battle scene emphasize the concept of the Golden Hour, which highlights the notion that for the patient “every minute counts” and for the emergency physician “every minute is borrowed time” in instances where control of the source of bleeding has not been achieved yet. Likewise, it is crucial to endorse a strategic plan in order to minimize any inexpedient delays in the management of patients with major bleeding. However, several gaps still exist in our knowledge regarding the overall treatment of patients with major bleeding, which hamper the optimal management of this vulnerable patient population. First and foremost, there is an absolute need for a universal definition of both major and critical bleeding, based on specific clinical criteria, which will enable their early recognition. Furthermore, it is of paramount importance to establish precise guidelines for the activation of massive transfusion protocols so as to guarantee their prompt implementation when indicated. Attention should also be paid to determining and validating appropriate transfusion strategies for each clinical scenario.

Apart from the need to address existing gaps, additional research is warranted in order to overcome certain controversies in the field of management of the bleeding patient. Therefore, the ideal type and volume of fluids for the initial resuscitation, along with the ideal transfusion ratio (1:1:1 or 1:1:2), the administration of FFP versus PCCs or other factor concentrates, the appropriate threshold of platelet transfusion, the use of tranexamic acid and Andexanet alfa, and the indicated approach to cirrhotic patients are issues that require further clarification.

Undoubtedly, the ED has a strategic role in the coordination of the multidisciplinary team involved in the bundles of care for the patient presenting with bleeding. In order to achieve the best possible results, it is imperative for the ED personnel to promote elaborate teamwork and establish a rigorous and smooth collaboration with the rest of the multidisciplinary healthcare alliance, which entails the prehospital team, the laboratory team, the surgical team and the radiology department. Hence, future research needs to focus on delineating multidisciplinary care practices that should aim to facilitate the patient’s pathway towards definite and optimal treatment.

## Figures and Tables

**Table 1:** Definitions of critical and major bleeding, major and massive haemorrhage and massive transfusion in the adult population, as per clinical guidelines.

Guidelines	Term	Definition
International Society on Thrombosis and Haemostasis, 2005 [2]	**Major bleeding**	**1.** Fatal bleeding **and/or ** **2.** Symptomatic bleeding in a critical area or organ, such as intracranial, intraspinal, intraocular, retroperitoneal, intra-articular or pericardial, or intramuscular with compartment syndrome **and/or** **3.** bleeding causing a fall in haemoglobin levels of 2 g/dL or more, or leading to a transfusion of ≥2 units of whole blood or RBCs
British Society for Haematology, 2022 [13]	**Major Haemorrhage**	Bleeding that leads to HR > 110 bpm **and/or** SBP < 90 mmHg
International Forum on the Management of Major Haemorrhage, 2022 [1]	**Massive Transfusion**	≥10 units of RBCs within 24 h (most common)
National Blood Authority of Australia, 2023 [14]	**Critical bleeding**	**1.** Major haemorrhage that is life-threatening and is likely to result in the need for massive transfusion (≥5 units of RBCs in 4 h) **2.** Haemorrhage of a smaller volume in a critical area or organ (e.g., intracranial, intraspinal or intraocular), resulting in patient morbidity or mortality.
Working Group on Emergency Disposition of Blood during a Red Phase Blood Shortage, Canadian National Advisory Committee on Blood and Blood Products, 2012 [15]	**Massive haemorrhage/Massive transfusion**	**1.** Expected blood loss of one BV over less than a 24-h period **OR ** **2.** 0.5 blood volume in 3 h **OR** **3.** ≥4 units of RBCs in one hour
Joint United Kingdom (UK) Blood Transfusion and Tissue Transplantation Services Professional Advisory Committee, 2018 [16]	**Major haemorrhage**	**1.** Loss of over 1 BV (70 mL/kg or >5 L in a 70 kg adult) in 24 h **OR** **2.** loss of 50% of TBV in <3 h **OR** **3.** bleeding at a rate >150 mL/min

BV = Blood Volume; bpm = beats per minute; HR = Heart Rate; RBCs = Red Blood Cells; SBP = Systolic Blood Pressure; TBV = Total Blood Volume.

**Table 2:** Recently proposed metrics to predict the need for massive transfusion and early mortality.

Metric	Definition	Authors, Year
**Critical Administration Threshold (CAT)**	≥3 units of RBCs in 1 h	Savage et al., 2015 [17]
**Resuscitation Intensity (RI)**	≥4 units of blood product (RBCs/plasma/crystalloid and colloid)	Meyer et al., 2018 [18]

RBCs = Red Blood Cells.

**Table 3:** Management of major bleeding in different clinical scenarios.

	MT (FFP:PLT:PRBC)	RBC	COAGULOPATHY (GUIDED BY LAB VALUES/VET)							
			FFP	FC/cryo	PCC	Platelets	Calcium	TXA	Other	Definite Treatment
**TRAUMA [4]**	1:1:1–1:1:2 FC/cryo:PLT:pRBC **OR ** FFP:PLT:pRBC Fibrinogen = 2 gr, FFP = 4 units, pRBC = 4 units	Target Hb 7–9 g/dL **OR** Hb 8 g/dL in CAD	15–20 mL/kg (if PT and/or APTT > 1.5 times normal [13]	3–4 g FC **OR** 15–20 single donor units of cryoprecipitate (if fibrinogen < 1.5 g/L OR guided by VET)	If fibrinogen is normal - >PCC 25–50 IU/kg (guided by VET)	Initial dose = 4–8 single PLT units **OR** 1 apheresis pack (target > 50 × 10^9^/L, >100 × 10^9^/L TBI)	CaCl_2_ (10 mL = 270 mg of elemental Ca^2+^)	TXA 1 g in 10 min within 3 h of trauma, 1 g over 8 h		Damage Control surgery
**UGIB**	1:1:2	Target Hb 7–9 g/dL **OR** Hb 8 g/dL in CAD [7,8,10,74,90,138]	*Caution* FFP↑ MORTALITY in CIRROTIC pts [90] - 12–15 mL/kg, if fibrinogen < 1 g/L **OR** INR/APTT > 1.5 [74,80] **OR** as indicated by coagulation tests [138]	25–50 mg/kg (if fibrinogen < 1.5 g/L) [74] or as indicated by coagulation tests [138]	20–30 IU/kg, as indicated by coagulation tests [74]	target > 50 × 10^9^/L [7,74,80] 4–6 single PLT units or 1 apheresis unit per 60–70 kg [74]	0.5–1.0 g per 500 mL of transfused blood [74]	Not recommended	**PPI** pantoprazole/esomeprazole 80 mg bolus, 8 mg/h [7,9,74,90] **ANTIBIOTIC** (ceftriaxone 1 g/24 h/erythromycin 250 mg IV 30–120 min before endoscopy) [7,74]	- Urgent Endoscopy in UNSTABLE pts or embolization if endoscopy fails [7,80] - Surgery
**LGIB**	1:1:2	1:1:2	As indicated by coagulation tests [138]	As indicated by coagulation tests [138]	20–30 IU/kg, as indicated by coagulation tests [74]	target > 50 × 10^9^/L [138]	0.5–1.0 g per 500 mL of transfused blood [74]	Not recommended		- Mesenteric embolization - Surgery
**PPH [13,73]**	1:1:1	Target Hb 7–9 g/dL **OR** Hb 8 g/dL in CAD	15–20 mL/kg (if PT and/or APTT > 1.5 times normal	Target > 2 g/L 10 single donor units of cryoprecipitate				- TXA 1 g in 10 min within 3 h-repeat if bleeding continues after 1 g [13]	- Oxytocin 10 IU IV, IF unavailable/ineffective: methyl-ergometrine [0.2 mg IM/IV slowly, every 2–4 h (max 5 doses)], or ergometrine 0.5 mg IM/IV slowly, or prostaglandin drug (sublingual misoprostol, 800 µg) [137]	- Manipulations: uterine massage, intrauterine balloon tamponade, uterine artery embolization - Damage Control Surgery
**HAEMOPTYSIS**								- TXA 1 g in 10 min within 3 h, 1 g over 8 h [39]	- Patient position to protect the well-aerated lung - CONSIDER early intubation if cough is not adequate	BAE
**MAJOR BLEEDING [13]**	1:2 FFP:pRBC	Target Hb 7–9 g/dL **OR** Hb 8 g/dL in CAD	15–20 mL/kg (if PT and/or APTT > 1.5 times normal	3–4 g FC (if fibrinogen < 1.5 g/L)		target > 50 × 10^9^/L				
**ICH [48]**	NA	NA	NA	NA	NA	target > 100 × 10^9^/L	NA		↓SBP 130–140 mmHg	

APTT = Activated Partial Thromboplastin Time; BAE = Bronchial Arteriography with Embolization; CAD = Coronary Artery Disease; Ca^2+^ = Ionized Calcium; cryo = Cryoprecipitate; FC = Fibrinogen Concentrate; FFP = Fresh Frozen Plasma; Hb = Haemoglobin; ICH = Intracranial Haemorrhage; IM = Intramuscularly; IV = Intravenously; INR = International Normalized Ratio; LAB = Laboratory; LGIB = Lower Gastrointestinal Bleeding; MT = Massive Transfusion; NA = Not Applicable; pRBC = packed Red Blood Cells; PT = Prothrombin Time; PCC = Prothrombin Complex Concentrate; PPH = Post-Partum Haemorrhage; PLT = Platelets; pts = patients; SBP = Systolic Blood Pressure; TBI = Traumatic Brain Injury; TXA = Tranexamic Acid; UGIB = Upper Gastrointestinal Bleeding; VET = Viscoelastic Tests.

**Table 4:** Reversal of anticoagulants in patients with major bleeding [139,140].

ANTICOAGULANTS	FFP		PCC	ANTIDOTES
**WARFARIN [140]**	15–30 mL/kg		25 IU/kg (INR 2–4) 35 IU/kg (INR 4–6) 50 IU/kg (INR > 6) max dose 100 IU	**vitamin K** 5–10 mg IV ***in concomitance**** with PCC/FFP* (because half-life of factor VII is only 6 h)
**DIRECT XA INHIBITORS**			25–50 IU/kg (If Andexanet alfa not available)	**Andexanet alfa** **low dose:** 400 mg (15 min), followed by 480 mg in 2 h, when: last dose of apixaban (≤5 mg)/rivaroxaban (≤10 mg) **≥ 8 h** last dose of rivaroxaban ≤ 10 mg **< 8 h/timing unknown** last dose of apixaban ≤ 5 mg **< 8 h/timing unknown**
				**high dose**: 800 mg (30 min), followed by 960 mg in 2 h, when: last dose of rivaroxaban > 10 mg/unknown dose **< 8 h** last dose of apixaban > 5 mg/unknown dose **< 8 h**
**DIRECT THROMBIN INHIBITORS**		20 single donor units of cryoprecipitate, if fibrinogen < 2 g/L [139]	25–50 IU/kg (max 4.000–5.000 IU) (if idarucizumab not available)	**Idarucizumab** 5 g (2 doses of 2.5 g with an interval of 15 min in between)
**UFH**				**Protamine** (max dose = 50 mg) - 1–1.5 mg/100 IU of UFH (if on IV UFH) - 0.5–0.75 mg/100 IU of UFH (if 30–60 min since last dose) - 0.25–0.375/100 IU of UFH (if >2 h since last dose)
**LMWH**				**Protamine** (max dose = 50 mg) - 1 mg of protamine for 1 mg enoxaparine (if <8 h after last dose) - 0.5 mg of protamine for 1 mg enoxaparine (if 8–12 h after last dose) - 1 mg of protamine for 100 units of dalteparin

IU = International Units; FFP = Fresh Frozen Plasma; LMWH = Low-Molecular-Weight Heparin; PCC = Prothrombin Complex Concentrate; Xa = Activated Factor X; UFH = Unfractionated Heparin.
